# Dynamic Akt/mTOR Signaling in Children with Autism Spectrum Disorder

**DOI:** 10.3389/fped.2017.00043

**Published:** 2017-03-15

**Authors:** Charity Onore, Houa Yang, Judy Van de Water, Paul Ashwood

**Affiliations:** ^1^The M.I.N.D. Institute, University of California Davis, Davis, CA, USA; ^2^Department of Medical Microbiology and Immunology, University of California Davis, Davis, CA, USA; ^3^Division of Rheumatology, Allergy and Clinical Immunology, University of California Davis, Davis, CA, USA

**Keywords:** autism, signaling, phosphorylation, T cells, mTOR

## Abstract

Autism spectrum disorder (ASD) is a behaviorally defined disorder affecting 1 in 68 children. Currently, there is no known cause for the majority of ASD cases nor are there physiological diagnostic tools or biomarkers to aid behavioral diagnosis. Whole-genome linkage studies, genome-wide association studies, copy number variation screening, and SNP analyses have identified several ASD candidate genes, but which vary greatly among individuals and family clusters, suggesting that a variety of genetic mutations may result in a common pathology or alter a common mechanistic pathway. The Akt/mammalian target of rapamycin (mTOR) pathway is involved in many cellular processes including synaptic plasticity and immune function that can alter neurodevelopment. In this study, we examined the activity of the Akt/mTOR pathway in cells isolated from children with ASD and typically developing controls. We observed higher activity of mTOR, extracellular receptor kinase, and p70S6 kinase and lower activity of glycogen synthase kinase 3 (GSK3)α and tuberin (TSC2) in cells from children with ASD. These data suggest a phosphorylation pattern indicative of higher activity in the Akt/mTOR pathway in children with general/idiopathic ASD and may suggest a common pathological pathway of interest for ASD.

## Introduction

Autism spectrum disorder (ASD) is characterized by severe and pervasive impairment in reciprocal social interaction skills, communication skills, or the presence of stereotyped behavior, interests, and activities (1). According to the most recent studies by the US Centers for Disease Control and Prevention, ASD is estimated to affect 1 in 68 children younger than 8 years (2). ASD remains a behaviorally defined disorder with no current physiological diagnostic tools or biological signatures. The cause(s) for the majority of cases of ASD remain unknown. In genetically identical monozygotic twins, there is a concordance rate of 44–91%; in dizygotic twins, the concordance rate for ASD is 0–37%; and in non-twin siblings, the rate is 0–24%; data that suggest a strong heritable component for this disorder (3–9). Although there is evidence to suggest that the disorder is highly heritable, no single genetic cause for all ASD has been identified. Heritability of ASD may suggest a genetic component in the disorder’s etiology; however, the genes involved vary greatly among individuals and family clusters and therefore suggest a more likely model that a variety of genetic mutations and/or environmental contributors may result in a common pathology or disruption of a common pathway.

Whole-genome linkage studies, genome-wide association studies, copy number variation screening, and SNP analyses have identified several ASD candidate genes (10). Associations between candidate genetic mechanisms and ASD have implicated a diverse range of functions including metabolism, immune function, neuronal migration, synapse formation, neuronal growth, and neurotransmission. Among some of the notable associations are mutations in *RELN* (11), *SHANK3* (12), *NLGN3, NLGN4X* (13), *MET* (14), *GABRB3* (15), *OXTR* (16), and *SLC6A4* (16). Furthermore, several single-gene mutation syndromic disorders incur increased risk of developing ASD including Rett syndrome (*MeCP2*), Fragile X (*FMR1*), Tuberous sclerosis (either *TSC1* or *TSC2*), Cowden syndrome (*PTEN*), Timothy syndrome (*CACNA1C*), and Angelman syndrome (*UBE3A*) (17–19). Even with the recent advancements in identifying candidate genes involved in ASD, all identified genetic risk factors combined account for only 10–20% of the total ASD population (10). The genetic mechanisms or mutations are clearly diverse and heterogeneous and may be influenced by environmental factors.

Among the potential candidate genes identified in ASD to date, those involved in Akt/mammalian target of rapamycin (mTOR) signaling and the downstream effects of this pathway are highly represented including *FMR1, PTEN, TSC1*, and *TSC2* (20). In addition, microarray analysis of peripheral blood suggests that there is abnormal activity in this pathway (21). The Akt/mTOR pathway is involved in many cellular processes that may impact neurodevelopment and have relevance to ASD symptoms. For example, in neurons, this pathway is believed to be important in the process of learning and memory formation by augmenting long-term potentiation (LTP) of synapses (22). The process of LTP involves the strengthening of synapses as a result of sustained signaling in the Akt/mTOR pathway (23).

The Akt/mTOR pathway is complex, and a defect in any of the proteins involved can lead to aberrant signaling. Increased Akt/mTOR activity is consistent with deficiencies of FMR1, TSC1/2, or PTEN found in Fragile X, TSC, and Cowden syndrome and suggests that increased Akt/mTOR activity may have a role in the pathophysiology of the general ASD population and not limited to single ASD genetic mutations. We hypothesized that there are a diverse collection of physiological abnormalities including genetic mutation or environment factors that converge to dysregulate the Akt/mTOR pathway in individuals with ASD.

To probe directly for dysregulation in the Akt/mTOR pathway, we examined the phosphorylation activity of several proteins in the Akt/mTOR pathway in children with ASD and typically developing (TD) controls. Protein phosphorylation was examined in freshly isolated T cells (24) and following stimulation from both ASD and TD control children of the same age. Phosphorylation levels of insulin receptor substrate-1 (IRS1), phosphatase and tensin homolog (PTEN), tuberin (*TSC2*), Akt, glycogen synthase kinase 3 (GSK3)α, GSK3β, mTOR, p70S6 kinase (p70S6K), ribosomal protein S6 (RPS6), and extracellular receptor kinase (ERK) were measured in T cells collected from peripheral blood of all subjects to cast a wide net over the entirety of the Akt/mTOR pathway. In this study, we describe a dysregulation of Akt/mTOR signaling in T cells isolated from children with ASD compared with TD control children, data that might help point to possible etiological mechanisms in ASD.

## Materials and Methods

### Subjects and Behavioral Assessments

Study participants were recruited as part of the Autism Phenome Project (25, 26). The study protocol was approved and carried out in accordance with the recommendations of the Institutional Review Board for the UC Davis School of Medicine, and parents of each subject provided written informed consent for their child to participate in accordance with the Declaration of Helsinki. Participants consisted of 41 children with ASD [mean age (SD) = 6.13 (1.23) years, 32 males] and 31 TD controls [mean age (SD) = 5.95 (1.27) years, 22 males] (Table 1). Age and male:female ratios were not statistically different. Diagnostic instruments for ASD included the Autism Diagnostic Observation Schedule-Generic (ADOS-G) (27) and the Autism Diagnostic Interview—Revised (28). ADOS scores [mean (SD)] were 12.6 (3.8) for social affect, 4.6 (1.7) for restricted/repetitive behaviors, and 7.4 (1.9) for severity. All diagnostic assessments were conducted or directly observed by trained, licensed clinical psychologists who specialize in autism and had been trained according to research standards for these tools. Inclusion criteria for ASD were taken from the diagnostic definition of ASD in young children formulated and agreed upon by the Collaborative Programs of Excellence in Autism. Inclusion criteria for TD controls included developmental scores within two SDs of the mean on all subscales of the Mullen’s scale of early learning. For ASD differential quotient (DQ) [mean (SD)] = 59.8 (27.5), verbal differential quotient (VDQ) = 72.3 (18.5), and non-verbal differential quotient (NVDQ) = 66.2 (21.7); for TD, DQ = 105.8 (11.4), VDQ = 105.8 (12.7), and NVDQ = 105.5 (10.1). Exclusion criteria for TD controls included any known developmental, neurological, or behavioral problems; a diagnosis of mental retardation, pervasive developmental disorder, or specific language impairment. TD children were screened and excluded for autism with the Social Communication Questionnaire (29) (scores > 11) (SCQ—Lifetime Edition). All participants were native English speakers, were ambulatory, and had no suspected vision or hearing problems. The exclusion criteria for all subjects consisted of the presence of Fragile X or other serious neurological (e.g., seizures), psychiatric (e.g., bipolar disorder), the presence of any known genetic polymorphism that causes ASD, or known medical conditions including autoimmune disease and inflammatory bowel diseases/celiac disease. All subjects were screened *via* parental interview for current and past physical illness. Children with known endocrine, cardiovascular, pulmonary, liver, or kidney disease were excluded from enrollment in the study. No participant presented with a cold, fever, or other common illness, if such a condition occurred, the blood draw were delayed until the child’s health status was stable for 48 h.

**Table 1:** **Study population demographics**.

Group	Autism spectrum disorder (ASD) (*N* = 41)	Controls (*N* = 32)
Age (years), median (range)	5.7 (4.6–9.3)	5.5 (4.5–9.9)
**Gender**		
Male	32 (72%)	22 (55%)
Female	9 (28%)	10 (45%)
**Race/ethnicity**		
Caucasian, non-Hispanic	26 (64%)	24 (75%)
African American, non-Hispanic	1 (2%)	–
Hispanic	8 (20%)	5 (16%)
Asian, non-hispanic	1 (2%)	–
Other, non-hispanic	5 (12%)	3 (9%)
**Mullen, median [interquartile range (IQR)]**		
Differential quotient (DQ)	56.1 (35.4–77.1)	105.8 (98.6–114.4)
Verbal differential quotient (VDQ)	69.1 (60.1–83.6)	105.8 (98.3–116)
Non-verbal differential quotient (NVDQ)	66.2 (48.4–78.9)	105.5 (99.2–112.1)
**Autism Diagnostic Observation Schedule (ADOS), median (IQR)**		
Social affect	12 (9–16)	N/A
Restricted and repetitive behavior	5 (3–6)	N/A
Severity	7 (6–9)	N/A

*Controls with a sibling or first-degree relative with ASD were excluded from the study. Behavioral data include Mullen’s Scales of Early Learning (DQ, VDQ, NVDQ) scores with median and interquartile range and ADOS (social affect, restricted and repetitive behavior, severity) scores with median and interquartile range*.

### Peripheral Blood Mononuclear Cell (PBMC) Collection

Peripheral blood was collected in acid-citrate-dextrose Vacutainers (BD Biosciences, San Jose, CA, USA) following behavioral assessments. Whole blood was centrifuged at 960 *g* for 10 min to separate cell fractions from plasma. The cell fraction was diluted in 25 ml of Hank’s Balanced Salt Solution (HBSS) (Mediatech, Manassas, VA, USA) and layered at room temperature over lymphocyte separation medium (LSM) (Mediatech) and centrifuged at 300 *g* for 30 min to isolate PBMCs. PBMCs were harvested at the interface between LSM and HBSS fractions and washed twice with 50 ml HBSS. The number of viable PBMC was determined by 1:1 dilution of Trypan Blue (Mediatech) exclusion counting on a Hausser phase contrast hemacytometer. Cells were then diluted in a chilled (4°C) magnetic separation buffer (MACS buffer) (Miltenyi Biotec, Auburn, CA, USA) to a concentration of 2.5 × 10^8^/ml.

### T Cell Isolation

T cells were isolated with a Pan T cell isolation kit according to the protocol provided by the manufacturer (Miltenyi Biotec). Briefly, a biotinylated antibody cocktail containing antibodies reactive to CD14, CD15, CD16, CD19, CD34, CD36, CD56, CD123, and CD235a (glycophorin A) was added at a concentration of 10 μl of antibody cocktail/10^7^ cells and incubated for 10 min at 4°C. Following incubation, an additional 30 μl of MACS buffer per 10^7^ cells was added followed by 10 μl of magnetic beads conjugated to antibiotin antibodies at a concentration of 20 μl/10^7^ cells and incubated at 4°C for an additional 15 min. Cells were then washed in 20-fold incubation volume of MACS buffer and centrifuged at 300 *g* for 10 min. Supernatants were aspirated and discarded, and PBMC were resuspended in 500 μl. This suspension was transferred to a MACS LS column on a MACS magnetic platform. The column was washed with a total of 9 ml MACS buffer, and eluent was again collected. All of the T cell-enriched eluent that passed through the LS column from the column was collected and centrifuged at 300 *g* for 10 min. The T cell-enriched pellet was washed and centrifuged twice in 10 ml of Roswell Park Memorial Institute (RPMI) media (Life technologies) containing 10% low endotoxin, heat-inactivated fetal bovine serum (Life technologies, Carlsbad, CA, USA), 100 IU/ml penicillin, and 100 IU/ml streptomycin (Sigma, St. Louis, MO, USA) (complete RPMI). The T cells were then resuspended in 1 × 10^6^ cell/ml and plated 1 ml/well in a 12-well sterile tissue culture plate (Greiner Bio-One, Monroe, NC, USA). T cells were allowed to rest in complete RPMI at 37°C for 16 h prior to stimulation and lysis.

### T Cell Stimulation, Lysis, and Protein Quantification

T cells were either left unstimulated or stimulated with 10 nM phorbol myristate acetate (PMA) for 15 or 45 min in complete RPMI at 37°C. Following stimulation, T cells were quickly washed in 10 ml of ice-cold PBS and centrifuged for 10 min at 500 *g* at 4°C. Supernatants were aspirated, and cell pellet was resuspended in ice-cold lysis buffer (Cell Signaling Technology, Danvers, MA, USA) containing 20 mM Tris-HCl (pH 7.5), 150 mM NaCl, 1 mM Na_2_EDTA, 1 mM EGTA, 1% Triton X, 2.5 mM sodium pyrophosphate, 1 mM beta-glycerophosphate, 1 mM Na_3_VO_4_, and 1 μg/ml leupeptin supplemented with freshly added 1% AEBSF. Cells were mixed in lysis buffer with vigorous pipetting and sonicated for 30 s. Cells were then incubated in lysis buffer on ice for 10 min. Following incubation, cell suspensions were centrifuged at 18,000 *g* for 10 min. Supernatants were collected, and aliquots were stored at −80°C until date of protein quantification or phosphorylation analysis. Protein levels were quantified using Bradford assay. In brief, diluted lysate and BSA standards were combined 1:1 with room temperature Bradford reagent (Bio-Rad, Hercules, CA, USA) and incubated for 10 min. Following incubation, optical density was measured with a 595 λ filter on a Bio-Rad spectrophotometer. Protein levels of T cell lysate samples were derived from a linear seven-point BSA standard curve (Bio-Rad).

### Phosphorylation Assay

Relative levels of phosphorylated and total IRS1, PTEN, ERK1/2, Akt, GSK3α, GSK3β, TSC2, mTOR, p70S6K, and RPS6 were measured with Milliplex™ multiplexing bead immunoassays (Millipore, Billerica, MA, USA). This assay contains antibodies that have been standardized and optimized for sensitivity and specificity to detect the phosphorylation and total protein levels of the molecules tested. Assay detection of the total and phosphorylated proteins detected was confirmed using Western blot analysis of individual phosphorylated or total proteins prior to testing, as well as the manufacturer’s own internal research quality controls. A subset of samples was also put through a second assay provided by Bio-Rad that contains many of standardized antibodies used in Western blot analyses, with strong correlation between assays (data not shown). Luminex technology provides a unique platform for analysis of limited sample volume, as is the case in this study, where precious blood samples from pediatric patients are used. GAPDH was used as an internal control for both phoshporylated and total protein assays, which were measured separately. The multiplex assay was performed according to the manufacturer’s instructions. Briefly, lysates were incubated with antibody-conjugated beads at a total protein concentration of 500 μg/ml. After protein capture, beads were washed twice followed by incubation with biotinylated detection antibody. Beads were then incubated with streptavidin-conjugated phycoerythrin (PE), followed by several washes. Finally, the bead sets were analyzed using a flow-based Luminex™ 100 suspension array system to determine bead identity and corresponding PE mean fluorescent intensity (MFI) (Bio-Plex 200; Bio-Rad Laboratories). Relative levels of phosphorylated and total proteins were determined by dividing MFI signal of each protein by GAPDH signal for each sample. Relative phosphorylation was determined by further dividing the GAPDH relative levels of phosphorylated proteins to their respective GAPDH relative total protein levels. All phosphorylation values are expressed as ratios.

### Statistical Analysis

The data were non-parametrically distributed, *p* values were determined with Mann–Whitney *U*-test. All analyses were two tailed, and values of *p* < 0.05 were considered statistically significant. For these novel and previously untested experiments, unadjusted *p* values are presented (30). All analyses were conducted using GraphPad Prism statistical software (GraphPad Software Inc., San Diego, CA, USA). Associations between Akt/mTOR/pathway measures and behaviors assessed by ADOS were calculated using a two-tailed, non-parametric Spearman’s correlation test with 95% confidence intervals.

## Results

Given that Akt/mTOR genetic mutations are potentially associated with increased ASD risk, we hypothesized that aberrations in many parts of the Akt/mTOR pathway will contribute to an overall pattern of increased Akt/mTOR pathway activity. To test this theory, we examined several proteins in the Akt/mTOR pathway. We observed increased IRS1 and RSP6 total protein in children with ASD compared with TD controls under unstimulated conditions (*p* < 0.03; Table 2). Similarly, total IRS1 and RSP6 were increased, in T cells following 15 or 45 min of stimulation, in the ASD group compared to TD controls (*p* < 0.04). No other total protein levels were different between groups (Table 2). For phosphorylated proteins, in unstimulated T cells, GSK3α, GSK3β, PTEN, TSC2, and mTOR were increased in children with ASD compared to TD controls (*p* < 0.006; Table 3). After 15 min of stimulation, T cells from children with ASD had higher phosphorylation of proteins, p7056K, IRS1, GSK3α, GSK3β, AKT, PTEN, TSC2, mTOR, and ERK (*p* < 0.04; Table 3). After 45 min of T cell stimulation, levels of phosphorylated protein were still increased in children with ASD for p7056K, IRS1, GSK3α, GSK3β, AKT, PTEN, TSC2, mTOR, and ERK, as well as RPS6 (*p* < 0.02; Table 3).

**Table 2:** **Akt/mTOR pathway total protein in T cells**.

Protein	ASD		Control		*p* Value
	Median	Interquartile range (IQR)	Median	IQR	
**Activating**					

**Unstimulated**					
Akt	0.175	0.132	0.169	0.088	0.708
Mammalian target of rapamycin (mTOR)	0.016	0.012	0.015	0.005	0.741
p70S6 kinase (p70S6K)	0.250	0.092	0.221	0.094	0.406
Ribosomal protein S6 (RPS6)	0.005	0.002	0.004	0.001	**0.017**
Extracellular receptor kinase (ERK)	0.655	0.149	0.646	0.076	0.551
**Stimulated (15 min)**					
Akt	0.173	0.124	0.171	0.085	0.792
mTOR	0.014	0.008	0.016	0.007	0.377
p70S6K	0.263	0.110	0.265	0.109	0.474
RPS6	0.006	0.004	0.005	0.002	**0.001**
ERK	0.604	0.196	0.605	0.155	0.669
**Stimulated (45 min)**					
Akt	0.168	0.107	0.169	0.090	0.853
mTOR	0.018	0.008	0.016	0.009	0.071
p70S6K	0.270	0.089	0.273	0.141	0.966
RPS6	0.014	0.016	0.009	0.008	**0.003**
ERK	0.607	0.115	0.617	0.118	0.664

**Inactivating**					

**Unstimulated**					
Insulin receptor substrate-1 (IRS1)	0.027	0.026	0.018	0.022	**0.030**
PTEN	0.289	0.156	0.255	0.079	0.129
Glycogen synthase kinase 3 (GSK) 3α	0.282	0.275	0.254	0.203	0.729
GSK3β	0.269	0.238	0.260	0.130	0.544
TSC2	0.040	0.049	0.039	0.056	0.590
**Stimulated (15 min)**					
IRS1	0.030	0.023	0.021	0.020	**0.041**
PTEN	0.307	0.146	0.266	0.075	0.067
GSK3α	0.209	0.245	0.280	0.214	0.053
GSK3β	0.153	0.169	0.168	0.143	0.378
TSC2	0.043	0.050	0.036	0.056	0.538
**Stimulated (45 min)**					
IRS1	0.040	0.028	0.024	0.031	**0.004**
PTEN	0.284	0.114	0.245	0.074	0.078
GSK3α	0.210	0.186	0.276	0.131	0.065
GSK3β	0.115	0.109	0.159	0.128	0.147
TSC2	0.040	0.049	0.035	0.056	0.866

*All p values were calculated with two-tailed Mann–Whitney U-tests*.

*Values in bold and gray shades signifies a p < 0.05*.

**Table 3:** **Akt/mTOR pathway phosphorylated protein in T cells**.

Protein	ASD		Control		*p* Value
	Median	Interquartile range (IQR)	Median	IQR	
**Activating**					

**Unstimulated**					
Akt	0.006	0.004	0.005	0.003	0.110
Mammalian target of rapamycin (mTOR)	0.024	0.017	0.015	0.012	**0.006**
p70S6 kinase (p70S6K)	0.011	0.007	0.008	0.004	0.064
Ribosomal protein S6 (RPS6)	0.009	0.006	0.009	0.005	0.473
Extracellular receptor kinase (ERK)	0.006	0.004	0.006	0.002	0.211
**Stimulated (15 min)**					
Akt	0.008	0.005	0.005	0.006	**0.013**
mTOR	0.099	0.088	0.057	0.088	**0.011**
p70S6K	0.093	0.142	0.027	0.125	**0.018**
RPS6	0.060	0.071	0.013	0.070	0.085
ERK	0.153	0.150	0.080	0.195	**0.029**
**Stimulated (45 min)**					
Akt	0.008	0.005	0.005	0.005	**0.006**
mTOR	0.127	0.094	0.071	0.107	**0.002**
p70S6K	0.142	0.140	0.069	0.145	**0.003**
RPS6	0.137	0.221	0.035	0.200	**0.023**
ERK	0.104	0.094	0.043	0.103	**0.005**

**Inactivating**					

**Unstimulated**					
Insulin receptor substrate-1 (IRS1)	0.005	0.004	0.004	0.003	0.064
PTEN	0.219	0.097	0.170	0.074	**0.000**
Glycogen synthase kinase 3 (GSK3)α	0.015	0.012	0.011	0.005	**0.002**
GSK3β	0.023	0.021	0.012	0.014	**0.000**
TSC2	0.016	0.012	0.013	0.006	**0.001**
**Stimulated (15 min)**					
IRS1	0.009	0.007	0.004	0.008	**0.009**
PTEN	0.208	0.119	0.175	0.111	**0.045**
GSK3α	0.317	0.326	0.161	0.361	**0.009**
GSK3β	0.116	0.099	0.064	0.118	**0.023**
TSC2	0.052	0.039	0.034	0.054	**0.027**
**Stimulated (45 min)**					
IRS1	0.010	0.016	0.005	0.006	**0.003**
PTEN	0.233	0.176	0.164	0.050	**0.001**
GSK3α	0.307	0.303	0.107	0.263	**0.001**
GSK3β	0.113	0.077	0.067	0.089	**0.002**
TSC2	0.054	0.046	0.028	0.039	**0.000**

*Median and IQR of phosphorylated Akt/mTOR pathway proteins. All p values were calculated with two-tailed Mann-Whitney U-tests*.

*Values in bold and gray shades signifies a p < 0.05*.

**Figure 1:**
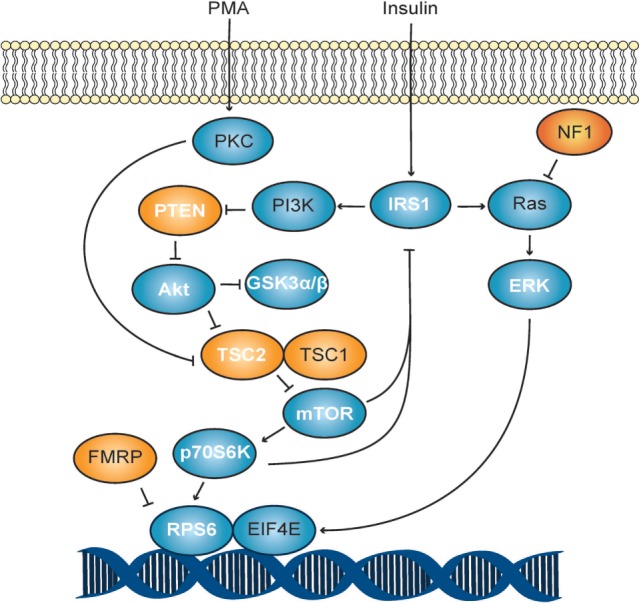
**Akt/mTOR signaling schematic**. The PI3K pathway in response to stimulation with phorbol myristate acetate (PMA). Autism spectrum disorder-associated mutations are shown in orange, while all others are shown in blue. Molecules measured in this study are shown with white lettering.

By calculating the ratio of phosphorylated to total protein levels, we observed higher ratios for mTOR and GSK3β (*p* < 0.02) in unstimulated ASD T cells when compared to unstimulated TD control T cells (Table 4; and Figures S1 and S2 in Supplementary Material). Given the effects of phosphorylation on these proteins (Table 5), it would indicate increased activity of mTOR and decreased activity of GSK3β in ASD T cells. This may suggest higher activity of Akt/mTOR signaling in ASD T cells (Figure 1). ASD T cells also trended toward increased phosphorylation of GSK3α under unstimulated conditions (Table 5), which would indicate lower activity of GSK3α consistent with higher Akt/mTOR pathway activity (Table 1). Together these data indicate Akt/mTOR signaling is higher in resting T cells of children with ASD when compared with T cells from TD controls.

**Table 4:** **Akt/mTOR pathway phosphorylated/total protein ratios in T cells**.

Protein	ASD		Control		*p* Value
	Median	Interquartile range (IQR)	Median	IQR	
**Activating**					

**Unstimulated**					
Akt	0.039	0.050	0.028	0.021	0.544
Mammalian target of rapamycin (mTOR)	1.262	1.529	0.941	0.866	**0.024**
p70S6 kinase (p70S6K)	0.041	0.038	0.035	0.019	0.254
Ribosomal protein S6 (RPS6)	1.685	1.532	1.995	1.395	0.590
Extracellular receptor kinase (ERK)	0.009	0.009	0.009	0.004	0.597
**Stimulated (15 min)**					
Akt	0.053	0.046	0.039	0.038	0.129
mTOR	5.954	6.491	2.770	5.730	**0.016**
p70S6K	0.342	0.653	0.104	0.357	**0.002**
RPS6	5.787	7.306	3.298	10.334	0.274
ERK	0.227	0.288	0.127	0.367	**0.027**
**Stimulated (45 min)**					
Akt	0.062	0.058	0.036	0.042	0.078
mTOR	6.769	6.872	3.929	5.815	**0.008**
p70S6K	0.503	0.588	0.225	0.417	**0.004**
RPS6	9.008	8.853	4.325	17.095	0.087
ERK	0.178	0.168	0.078	0.186	**0.050**

**Inactivating**					

**Unstimulated**					
Insulin receptor substrate-1 (IRS1)	0.235	0.294	0.250	0.224	0.346
PTEN	0.681	0.461	0.647	0.278	0.115
Glycogen synthase kinase 3 (GSK3)α	0.072	0.283	0.043	0.090	0.057
GSK3β	0.073	0.111	0.050	0.042	**0.010**
TSC2	0.428	2.717	0.360	1.653	0.153
**Stimulated (15 min)**					
IRS1	0.305	0.695	0.275	0.347	0.834
PTEN	0.680	0.452	0.673	0.276	0.636
GSK3α	1.409	6.295	0.505	1.964	**0.004**
GSK3β	0.808	0.921	0.484	0.969	0.055
TSC2	1.797	7.840	0.867	2.747	**0.039**
**Stimulated (45 min)**					
IRS1	0.232	0.374	0.254	0.352	0.874
PTEN	0.860	0.445	0.663	0.207	**0.025**
GSK3α	1.199	4.969	0.364	1.290	**0.004**
GSK3β	0.977	0.783	0.523	0.899	**0.017**
TSC2	1.548	5.008	0.786	1.909	**0.029**

*Median and IQR of phosphorylated/total Akt/mTOR pathway proteins. All p values were calculated with two-tailed Mann–Whitney U-tests*.

*Values in bold and gray shades signifies a p < 0.05*.

**Table 5:** **Effect of phosphorylation on target sites of Akt/mTOR pathway proteins**.

Activating		Inactivating	
Akt	Ser473	IRS1	Ser312
mTOR	Ser2448	PTEN	Ser380
p70S6K	Thr412	GSK3α	Ser21
RPS6	Ser235/Ser236	GSK3β	Ser9
ERK	Thr185/Tyr187	TSC2	Ser939

*Proteins are organized according to whether the effect of phosphorylation on a specific phosphorylation site is activating or inactivating. The sites listed in the table are those measured in this article*.

After stimulation with PMA for 15 min, T cells from children with ASD exhibited higher phosphorylation of ERK, mTOR, p70S6K, GSK3α, and TSC2 compared with T cells from children with TD (*p* < 0.04; Table 4), indicating increased activity of ERK, mTOR, and p70S6K but a decreased activity of inhibitory signals by TSC2 and GSK3α, suggesting that Akt/mTOR pathway activity may be increased in stimulated ASD T cells (Table 1). ASD T cells also trended toward increased phosphorylation of GSK3β (*p* < 0.054), which would indicate lower activity of inhibitory GSK3β consistent with higher Akt/mTOR pathway activity. After 45 min of PMA stimulation, increased phosphorylation of ERK, mTOR, p70S6K, GSK3α, GSK3β, PTEN, and TSC2 were detected in ASD T cells (Table 4). There was also a trend for increased AKT but which did not quite reach statistical significance (*p* = 0.077). Together these data suggest overall increased AKT/mTOR pathway activity in ASD T cells following stimulation.

Associations were observed for total p7056k and autism severity at 15 min poststimulation (*r* = 0.327, *p* = 0.04). Restrictive and repetitive behaviors were associated with the PTEN ratio after 15 min stimulation also (*r* = −0.3316, *p* = 0.03). For social affect, several measures were associated including total p7056k and the IRIS ratio in unstimulated and 45 min after stimulation (*p* < 0.05).

## Discussion

In this study, we report differential activity of several Akt/mTOR signaling molecules in young children with ASD. To observe dynamic phosphorylation activity, freshly isolated T lymphocyte cells were chosen as a cellular representative that could be acquired efficiently, safely, and easily from relatively non-invasive blood samples. From our experiments, we determined that ASD T cells generally exhibit phosphorylation to total protein ratios that would indicate higher activity of mTOR, ERK, and p70S6K as well as lower activity of GSK3α, GSK3β, TSC2, and PTEN than TD control T cells. This indicates a shift toward higher Akt/mTOR pathway activity in the ASD group (Table 5; Figure 1). An increased Akt/mTOR activity is consistent with deficiencies of FMR1, TSC1/2, or PTEN found in Fragile X, TSC, and Cowden syndrome, respectively (31–33). Moreover, suppression of this increased Akt/mTOR activity has been demonstrated to improve ASD-associated symptoms in mice deficient for PTEN and TSC1 (34, 35). Together these data suggest that increased Akt/mTOR activity may have a role in the pathophysiology of the general ASD population and not limited to known ASD-associated Akt/mTOR genetic mutations.

The Akt/mTOR pathway is involved in a large number of physiological functions, in both the central nervous and immune systems (36–39). Atypical Akt/mTOR signaling may be related to many previous observations of abnormal T cell function (40–44) in children with ASD. The aberrancies in Akt/mTOR signaling observed in this study are likely not limited to T cells but will have relevance to signaling also in other immune cells and as such these data have relevance to other immune abnormalities previously observed in ASD involving multiple leukocyte subsets (25, 45–50). Aberrant Akt/mTOR signaling has the potential to impact cellular growth, proliferation, and cytokine production in the immune system (38), which can in turn affect behavior (26).

Our data show that immune dysfunction of children with ASD previously demonstrated may stem from aberrant T cells signaling *via* the Akt/mTOR pathway. To probe directly for dysregulation in the Akt/mTOR pathway, we sought to examine the phosphorylation activity of several proteins in the Akt/mTOR pathway in children with ASD and TD controls. As ASD manifests in early childhood, it is difficult to find suitable research tools and accessible tissues for experimentation. For example, postmortem human tissue can never provide the substrate for dynamic functional studies, and finding suitable control material is problematic. Immune cells, in contrast, provide a readily accessible model system that has many advantages including easy acquisition, high availability, and fine matching with controls. The advantages of using lymphocytes as an easily accessible “neural probe” in the investigation of psychiatric disorders in living subjects has been previously reviewed (24). We therefore utilized T lymphocytes as a neuronal surrogate in our experiments to examine dynamic signaling activity. T lymphocyte cells were chosen as a cellular model in which to test Akt/mTOR pathway activity for several characteristics including their long life span and high numbers within the blood. Moreover, the Luminex technology was chosen as it provides a platform for analyses of a number of analytes simultaneously from small volumes of tissue. As this study utilized pediatric blood samples, T cell numbers were limited, and thus, performing Western blot analyses on all phosphorylated or total proteins would be prohibitive. Importantly, the antibodies used had been previously standardized and optimized, and we also checked for detection of the same proteins in Western blot analysis and intracellular flow cytometry techniques, using Jurkat cells and primary T cells from adults, prior to running the Luminex assays. In addition, on a subset of samples, a number of phosphorylated or total proteins were compared between two Luminex assays from different manufacturer’s, with similar results. Our results suggest that the Luminex platform provides a quick and efficient means of identifying possible changes in the Akt/mTOR pathway, in pediatric samples that are limited in volume. Although our data showed increased Akt/mTOR signaling in ASD, whether this reflects what happens *in vivo* or within other tissues such as the gastrointestinal tract or brain is not known and would need further investigation.

Further work needs to be performed to determine context-dependent effects on Akt/mTOR pathway in T cells and how they relate to the brain; however, many gene expression studies have taken the approach to look at primary or immortalized blood cells as a surrogate for inaccessible tissue such as the brain. The advantages of using lymphocytes as neural surrogates for *in vitro* examination has been previously established, but there is also evidence that the increased Akt/mTOR activity observed in Fragile X central nervous system (CNS) tissue is mirrored in lymphocytes (51), suggesting that Akt/mTOR signaling in T cells is applicable to cells of the CNS, including neurons and glial cells. In neurons, the Akt/mTOR pathway is essential in the regulation of dendritic arborization and spine formation (52), which are important features of synapse formation. Increased activity of this pathway in neuronal knockouts of *TSC1* or *PTEN* results in lower sociability and seizures in mouse models (53–55), suggesting that both sociability and seizures are Akt/mTOR pathway activity dependent. Increased activity of this pathway in glial cells can also have negative effects on neurobiology, such as aberrant neuronal organization and seizures in astrocyte-specific *TSC1* conditional knockout mice (56). Lack of social interactions is a central symptom of ASD (1), and seizures are a common comorbidity in the disorder (57). Together these data suggest that these ASD symptoms could be potentially related to the high Akt/mTOR signaling as described in this study in ASD cells (53, 56, 58).

Akt activation leads to a number of effects, some of which are mTOR independent. These include regulating cell survival and growth, such as phosphorylation of Forkhead box O family of transcription factors and of GSK3α and GSK3β (59–61). GSK3 exists in two genetically distinct isoforms but with near identical function. GSK3α and GSK3β share 85% amino acid identity and 98% amino acid sequence homology within their kinase domains (62). The GSK3 proteins are involved in regulating metabolic function and are phosphorylated and inhibited by Akt among other kinases such as p70S6K. Once phosphorylated, the kinase activity of GSK3 is inhibited, and their substrates such as glycogen synthase, Ap-1, β*-*catenin, c-myc, and p53, thereby initiating signaling mechanisms promoting cell survival and growth. GSK3s are expressed in virtually all cell types, but their expression is highly enriched in the CNS (63) and appears to be involved in regulating synaptic plasticity (64). These proteins have recently gained attention in the area of Alzheimer’s disease (AD) research, and there have been several observations that both the activity and total levels of GSK3 is upregulated in AD patients (65). Interestingly, we observed lower GSK3 activity in children with ASD compared with TD controls. Current evidence suggests that low GSK3 activity impairs LTD (66), which could affect synaptic plasticity and in children with ASD.

Collectively, these data described in this study suggest a general dysregulation of the Akt/mTOR pathway in an idiopathic ASD population. This may suggest a convergent pathology in ASD that would affect multiple physiological symptoms. It is unclear whether Akt/mTOR aberrancies described in the study are due to as yet unknown genetic mutations, epigenetic changes, or environmental factors, and it is possible that it may be due to a combination of genetic and environmental influences. In fact, growth factors that imbue effects by signaling through the Akt/mTOR pathway such as HGF and MIF have also been reported as dysregulated in ASD (67, 68), suggesting that circulatory homeostatic factors can be an additional source of Akt/mTOR pathway activity. Similarly, a mutation in cMET, the receptor for HGF, has also been reported in ASD (69), suggesting that Akt/mTOR-associated receptors may also be a source for aberrant signaling activity. Together, these data present a novel finding Akt/mTOR pathway dysregulation in young children with ASD that could provide a focus for targeted therapeutics for at least a subset of individuals with ASD.

## Author Contributions

All authors designed the experiments, helped with data analysis and interpretation, and played a major role in writing the manuscript.

## Conflict of Interest Statement

The authors declare that the research was conducted in the absence of any commercial or financial relationships that could be construed as a potential conflict of interest.

## Supplementary Material

Figure S1 **Akt/mammalian target of rapamycin (mTOR) pathway activating phosphorylated/total protein ratios in T cells**. Box and whisker plots show activating AKT/mTOR pathway (phosphorylated/total) protein levels after 0, 15, and 45 min of stimulation between autism spectrum disorder (ASD) (black bars) and controls (gray bars). Data are depicted as box showing the lower (25%) and upper (75%) quartiles and the middle line representing the median, whiskers showing the minimum and maximum. **p* Value less than 0.05, calculated with two-tailed Mann–Whitney *U*-tests.

Figure S2 **Akt/mammalian target of rapamycin (mTOR) pathway inactivating phosphorylated/total protein ratios in T cells**. Box and whisker plots show inactivating AKT/mTOR pathway (phosphorylated/total) protein levels after 0, 15, and 45 min of stimulation between autism spectrum disorder (ASD) (black bars) and controls (gray bars). Data are depicted as box showing the lower (25%) and upper (75%) quartiles and the middle line representing the median, whiskers showing the minimum and maximum. **p* Value less than 0.05, calculated with two-tailed Mann–Whitney *U*-tests.
