# Supplementary figures and images for: Dynamic Akt/mTOR Signaling in Children with Autism Spectrum Disorder
Source: Front Pediatr. 2017 Mar 15;5:43. doi: 10.3389/fped.2017.00043 (PMC5350147; doi:10.3389/fped.2017.00043)

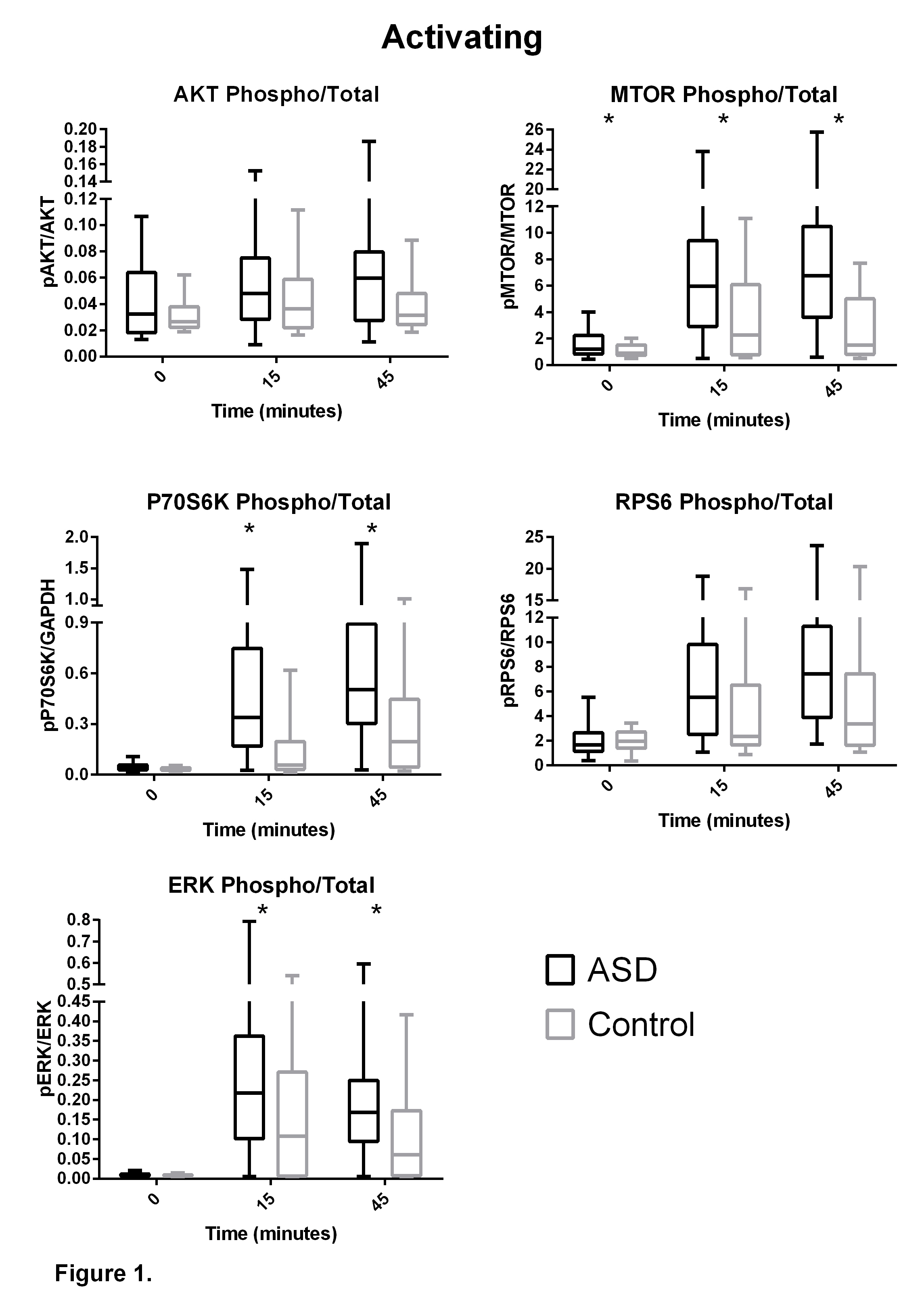

Supplement: Figure S1 — Akt/mammalian target of rapamycin (mTOR) pathway activating phosphorylated/total protein ratios in T cells. Box and whisker plots show activating AKT/mTOR pathway (phosphorylated/total) protein levels after 0, 15, and 45 min of stimulation between autism spectrum disorder (ASD) (black bars) and controls (gray bars). Data are depicted as box showing the lower (25%) and upper (75%) quartiles and the middle line representing the median, whiskers showing the minimum and maximum. *p Value less than 0.05, calculated with two-tailed Mann–Whitney U-tests. [file Image_1.JPEG]

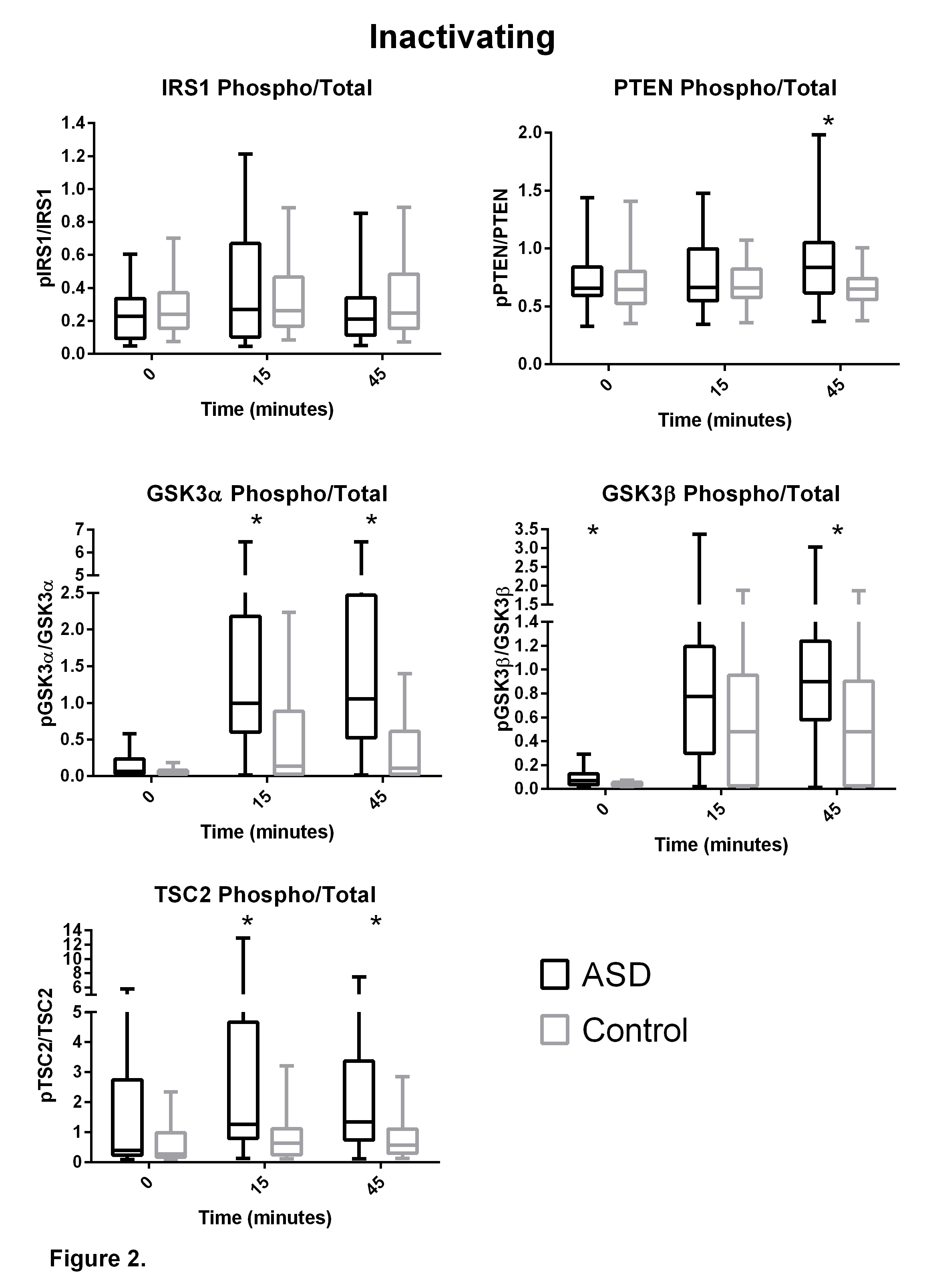

Supplement: Figure S2 — Akt/mammalian target of rapamycin (mTOR) pathway inactivating phosphorylated/total protein ratios in T cells. Box and whisker plots show inactivating AKT/mTOR pathway (phosphorylated/total) protein levels after 0, 15, and 45 min of stimulation between autism spectrum disorder (ASD) (black bars) and controls (gray bars). Data are depicted as box showing the lower (25%) and upper (75%) quartiles and the middle line representing the median, whiskers showing the minimum and maximum. *p Value less than 0.05, calculated with two-tailed Mann–Whitney U-tests. [file Image_2.JPEG]
